# miR-365 targets β-arrestin 2 to reverse morphine tolerance in rats

**DOI:** 10.1038/srep38285

**Published:** 2016-12-06

**Authors:** Jian Wang, Wei Xu, Tao Zhong, Zongbin Song, Yu Zou, Zhuofeng Ding, Qulian Guo, Xinzhong Dong, Wangyuan Zou

**Affiliations:** 1 Department of Anesthesiology, Xiangya Hospital, Central South University, Changsha, Hunan 410008, China; 2 The Solomon H. Snyder Department of Neuroscience, Johns Hopkins University, School of Medicine, Baltimore, Maryland 21205, USA; 3 Howard Hughes Medical Institute, Johns Hopkins University School of Medicine, Baltimore, Maryland 21205, USA

## Abstract

Morphine tolerance is a challenging clinical problem that limits its clinical application in pain treatment. Non-coding microRNAs (miRNAs) modulate gene expression in a post transcriptional manner, and their dysregulation causes various diseases. However, the significance of miRNAs in morphine tolerance is still poorly understood. In the present study, we hypothesized that microRNA-365 (miR-365) is a key functional small RNA that reverses morphine tolerance through regulation of β-arrestin 2 in rats. Here, microarray analysis and quantitative real-time PCR showed that miR-365 was robustly decreased in the spinal cord after chronic morphine administration. *In situ* hybridization and immunochemistry double staining showed that miR-365 was expressed in neurons of the spinal cord. We identified β-arrestin 2 as the target gene of miR-365 by bioinformatics analysis and luciferase reporter assay. The data showed that overexpression of miR-365 prevented and reversed established morphine tolerance, and increased expression of miR-365 caused a decrease in expression of β-arrestin 2 protein. miR-365 downregulation is involved in the development and maintenance of morphine tolerance through regulation of β-arrestin 2, and miR-365 upregulation provides a promising and novel approach for treatment of morphine tolerance.

Morphine is commonly used in clinical management to alleviate moderate to severe pain. However, prolonged and repeated use of morphine leads to tolerance^1^. In some patients, even the maximum tolerated dose of morphine cannot achieve a sufficient analgesic effect^2^,^3^. Morphine tolerance is defined as a gradual loss of drug potency or efficacy and reduced duration of action. When morphine tolerance occurs, dose escalation becomes necessary to maintain the same analgesic effect, which results in an increased likelihood of side effects. Thus far, the mechanisms underlying morphine tolerance are poorly understood. Multiple factors may be involved in these complex processes, such as opioid receptor desensitization^4^, changes in glutamate receptor function^5^,^6^, protein kinase C (PKC) activation^6^, and G-protein uncoupling^7^. In addition, some studies reported that miRNAs play a vital role in regulating morphine tolerance and chronic pain^8^,^9^,^10^.

miRNAs bind to 3′-UTRs (untranslated region) of target gene mRNA, inhibiting or destabilizing translation of the transcripts^11^,^12^,^13^. miRNAs have been shown to modulate expression of various genes in different systems^14^,^15^,^16^,^17^. Recent studies have found miRNAs are also involved in perioperative medicine, including inflammation, ischemia, sepsis and mediating anesthetic toxicity^18^,^19^,^20^,^21^,^22^. However, the specific role of miRNAs in morphine tolerance is still poorly understood.

Studies have reported that miRNAs are highly expressed in the CNS where opioid activity occurs^23^,^24^,^25^,^26^, and some are involved in morphine tolerance, such as fentanyl increased NeuroD protein level was mediated by miR-190^8^. He *et al*.^10^ have shown that the μ opioid receptor (MOR) mRNA 3′-UTR has a binding site for let-7 and that let-7 could repress MOR expression in mice. Inhibition of let-7 can alleviate morphine tolerance. Wu *et al*.^27^ found that miRNA-23b could repress MOR translation by interacting with the K box motif in the 3′-UTR of MOR 1, and the interaction could suppress receptor translation by blocking polysome-mRNA association. Morphine also decreased miR-133b expression and affected dopaminergic neuron differentiation in zebrafish embryos^28^. Therefore, the roles of miRNAs in regulating morphine tolerance are of growing interest.

Here we present evidence that miR-365 is involved in the modulation of morphine tolerance. In the study, we focused on miR-365 for several reasons: Firstly, miR-365 is expressed in the CNS and involved in neurological disorders^29^, suggesting a potential role of miR-365 in CNS dysfunction. Secondly, based on miRNA microarray profiling, miR-365 was robustly down-regulated in the spinal cord after chronic administration of morphine. Thirdly, β-arrestin 2, which is an important modulator of morphine tolerance^30^, is the predicted target of miR-365, and there is little information concerning how miR-365 regulates development of morphine tolerance. Therefore, we designed the study to investigate the role of miR-365 in regulating morphine tolerance. We hypothesized that miR-365 in the spinal cord regulates development and maintenance of morphine antinociceptive tolerance by targeting the G-protein-coupled receptor (GPCR) binding protein β-arrestin 2.

## Results

### miR-365 is down-regulated in the spinal cord of morphine-tolerant rats

**Figure 1:**
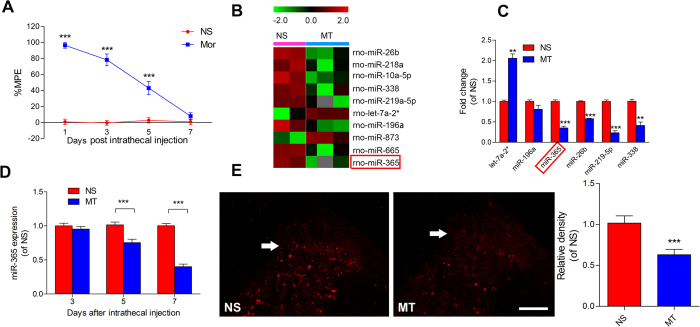
Downregulation of miR-365 in the spinal cord after induction of morphine tolerance. (**A**) Development of tolerance to morphine-induced antinociception assessed by the tail flick test. Tail flick latency was converted to %MPE. Values are expressed as mean ± s.e.m., n = 6, ***P < 0.001 compared with the normal saline group by two-way repeated-measures ANOVA followed by Bonferroni correction. (**B**) Heat maps of microRNAs shown by microarray analysis to be significantly upregulated or downregulated in the L4 ~ 5 spinal cord of rats following intrathecal injection of morphine compared with normal saline. The heat map diagram shows the result of log2 value of each miRNA microarray signal in different conditions (morphine or saline injection). Each row represents a miRNA and each column represents a sample. Color scale is shown on the top. (**C**) Representative examples of microRNAs showing upregulation or downregulation following independent verification with qRT-PCR analyses, n = 3. **P < 0.01, ***P < 0.001 relative to the normal saline group by unpaired t-test. (**D**) Expression level of miR-365 in the L4 ~ 5 spinal cord after chronic injection of morphine. Data are expressed as mean ± s.e.m., n = 6, ***P < 0.001, versus the normal saline group by two-way repeated-measures ANOVA followed by Bonferroni correction. (**E**) *In situ* hybridization images of miR-365 in the spinal cord from normal saline and morphine tolerance groups. n = 4, ***P < 0.001, compared with the normal saline group by Student’s *t*-test. Scale bar = 200 μm. Each experiment was performed in duplicate with the indicated number of samples. MT = morphine tolerance, NS = normal saline, miR = microRNA, MPE = maximum possible effect.

To identify miRNAs responsible for morphine tolerance, we profiled miRNA expression patterns through miRNA microarray analysis using L4 ~ 5 of spinal cords isolated from rats subjected to injection of morphine for 7 consecutive days, which is a classic model to induce morphine tolerance. Morphine tolerance was already established 7 days after morphine injection (Fig. 1A). Compared with the normal saline (NS) group (Fig. 1B), we found a broad range of miRNA expression changes in the morphine treated (MT) spinal cord. miR-365 was one of the most robustly reduced of these miRNAs, and the expression levels of miR-365 were confirmed by qPCR (Fig. 1C). miR-365 expression was not altered on day 3 after morphine injection, however, on day 5 it began to decline (Fig. 1D), and these changes were consistent with development of morphine tolerance. In addition, among the target genes of miR-365, some target genes participate in morphine tolerance (described in the discussion). For this reason, miR-365 was studied further to determine its role in morphine tolerance.

**Figure 2:**
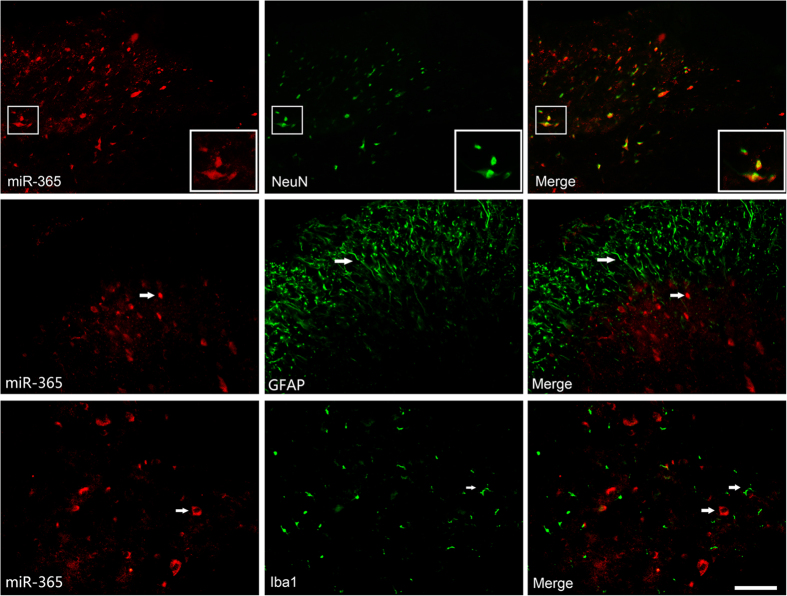
Cell-specific localization of miR-365 in the spinal cord. Cell-specific localization of miR-365 in the spinal cord as assessed by *in situ* hybridization with subsequent coimmunofluorescence staining of the spinal cord for miR-365 (red), neurons using NeuN antibody (green), astrocytes using GFAP antibody (green), and microglia using Iba1 antibody (green). Scale bar = 50 μm.

To explore further the localization and expression of miR-365 in the spinal cord, miRNA-specific *in situ* hybridization and staining were employed. The data showed that miR-365 was expressed in the dorsal horn of the spinal cord. Compared with the normal saline group, miR-365 expression was decreased on day 7 after morphine administration (Fig. 1E). The result was consistent with previous quantitative RT-PCR analysis. Co-immunostaining revealed high expression of miR-365 in the neuron specific NeuN-positive neurons of the spinal dorsal horn (Fig. 2).

### Lentivirus-mediated overexpression of miR-365 prevents and reverses morphine tolerance in rats

**Figure 3:**
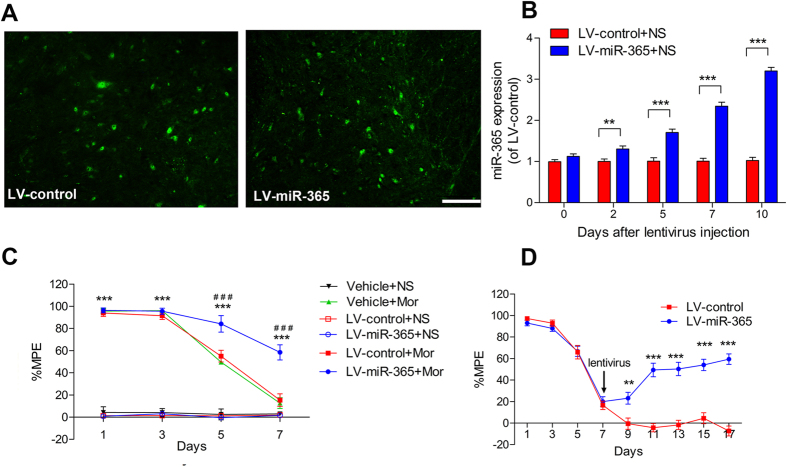
Lentiviral-mediated overexpression of miR-365 prevents and reverses morphine tolerance in rats. (**A**) Representative image of enhanced green fluorescent protein immunofluorescence in the spinal cord on day 7 after injection of LV-miRNA-365 or LV-control vector. Scale bar = 100 μm. (**B**) miR-365 expression levels in the L4 ~ 5 spinal cord after lentivirus injection. Rats were injected with control or miR-365 lentivirus vector 3 days before chronic normal saline infusion. Values are expressed as percentage of values of the LV-control + NS group, n = 6, ***P < 0.001 by two-way repeated-measures ANOVA followed by Bonferroni test. (**C** and **D**) Effect of injection of lentivirus miR-365 on the development of morphine tolerance. (**C**) Groups of rats were pretreated with vehicle (normal saline), LV-control vector, or LV-miR-365 vector 3 days before chronic injection of morphine or saline. Data are presented as mean ± s.e.m, n = 6, ***P < 0.001 compared with the Vehicle + NS group. ^###^*P* < 0.001 compared with the LV-control + Mor group by two-way repeated-measures ANOVA followed by Bonferroni test. (**D**) Groups of rats were injected with morphine twice a day for 17 consecutive days. LV-control vector or LV-miR-365 vector was administered on day 7 after establishment of morphine tolerance. Data are presented as mean ± s.e.m., n = 6, **P < 0.01, ***P < 0.001 compared with the LV-control group by two-way repeated-measures ANOVA followed by Bonferroni test. Each experiment was performed in triplicate with the indicated number of samples. miR = microRNA, MPE = maximum possible effect, NS = normal saline, Mor = morphine.

To confirm delivery of lentivirus (LV)-miR-365 or LV-control into neuronal cells of the spinal cord, transfected cells in the spinal cord were visualized by GFP fluorescence. After intrathecal administration of LV-miR-365 or LV-control, we found that many neuronal cell bodies were highly fluorescent, suggesting that the lentivirus was successfully transfected into neuronal cells (Fig. 3A).

miR-365 expression was gradually increased above basal levels 5 days after intrathecal LV-miR-365 injection and remained increased 10 days after treatment with LV-miR-365 (Fig. 3B). To explore the role of miR-365 in modulation of morphine tolerance at the behavioral level, we first pretreated rats with intrathecal injection of LV-miR-365, LV-control vector, or vehicle (normal saline) 3 days before chronic morphine or normal saline infusion. The basal latency of tail flick test was not changed in all groups (data were not shown). Rats treated with saline infusion (Vehicle + NS, LV-control + NS and LV-miR-365 + NS) showed no significant difference in %MPE during the 7 days. In rats treated with morphine infusion, rats pretreated with normal saline (Vehicle + Mor) showed 95.6 ± 5.4% (assessed by %MPE) antinociception on day 1; however, from day 3 to day 7, %MPE was significantly reduced compared with day 1. In contrast, rats pretreated with LV-miR-365 maintained antinociception through day 7 after chronic morphine injection and showed a significant increase in %MPE compared with rats treated with normal saline. Rats pretreated with LV-control vector (LV-control + Mor) had a %MPE similar to the Vehicle + Mor group (Fig. 3C). Then we post-treated rats with LV-control or LV-miR-365 vector on day 7 after establishment of morphine tolerance. We found that overexpression of miR-365 significantly reversed morphine tolerance (Fig. 3D). These results suggest that miR-365 in the spinal cord contributes to modulation of morphine tolerance.

### miR-365 directly targets β-arrestin 2

**Figure 4:**
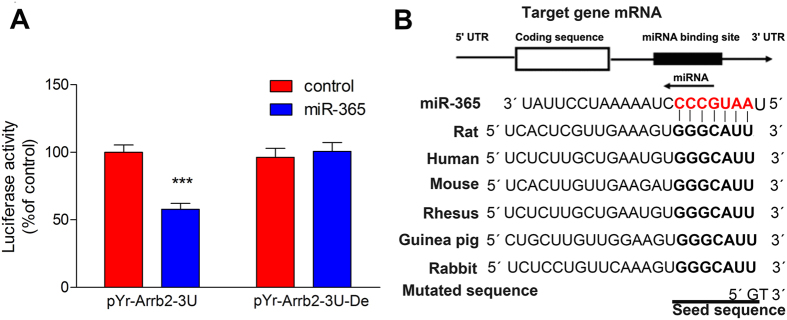
miR-365 directly targets the β-arrestin 2 3′-UTR. (**A**) Activity of luciferase with the β-arrestin 2 3′-UTR or a deletion mutation in the 3′-UTR in HEK293T cells cotransfected with control microRNA or a miR-365 mimic (n = 3). Values are expressed as percentage of values of the control group, ***P < 0.001 by Student’s *t* test. Each experiment was performed in triplicate with the indicated number of samples. (**B**) Schematic representation of the miR-365 sequence and its target sequence within the β-arrestin 2 3′-UTR. The target sequence is well conserved among mammals. The seed sequence is indicated by bold letters, while the pairing sequence of miR-365 is in red bold letters. miR = microRNA, UTR = untranslated region, Del = deletion mutation.

To identify target genes of miR-365 relevant to morphine tolerance, we searched for potential candidate targets using bioinformatics tools: TargetScan, miRanda, and miRDB. Among the putative genes identified, we focused on β-arrestin 2 because it plays a vital role in morphine tolerance^30^,^31^,^32^,^33^. Mice with a β-arrestin 2 null mutation are less likely to develop morphine tolerance^30^. Therefore, we examined whether β-arrestin 2 is a direct target of miR-365 using a luciferase assay. The 3′-UTR sequence (including the predicted mir-365 target sequence) of β-arrestin 2 was cloned into a reporter vector downstream of the *Renilla* luciferase gene. After cotransfection of HEK293 cells with the reporter vector containing miR-365, luciferase activity with the β-arrestin 2 3′-UTR was attenuated (Fig. 4A). To identify further the miR-365 binding sequence within the 3′-UTR, we mutated the predicted seed sequence with a partial deletion (Fig. 4B). We found that miR-365 had no effect on luciferase activity (Fig. 4A). These findings suggest that miR-365 directly targets the β-arrestin 2 3′-UTR in a sequence-specific manner. Furthermore, Fig. 4B shows that the miR-365 binding site is well conserved among mammals, which indicates that β-arrestin 2 regulation by miR-365 is functionally important.

### β-arrestin 2 is responsible for miR-365-mediated morphine tolerance

**Figure 5:**
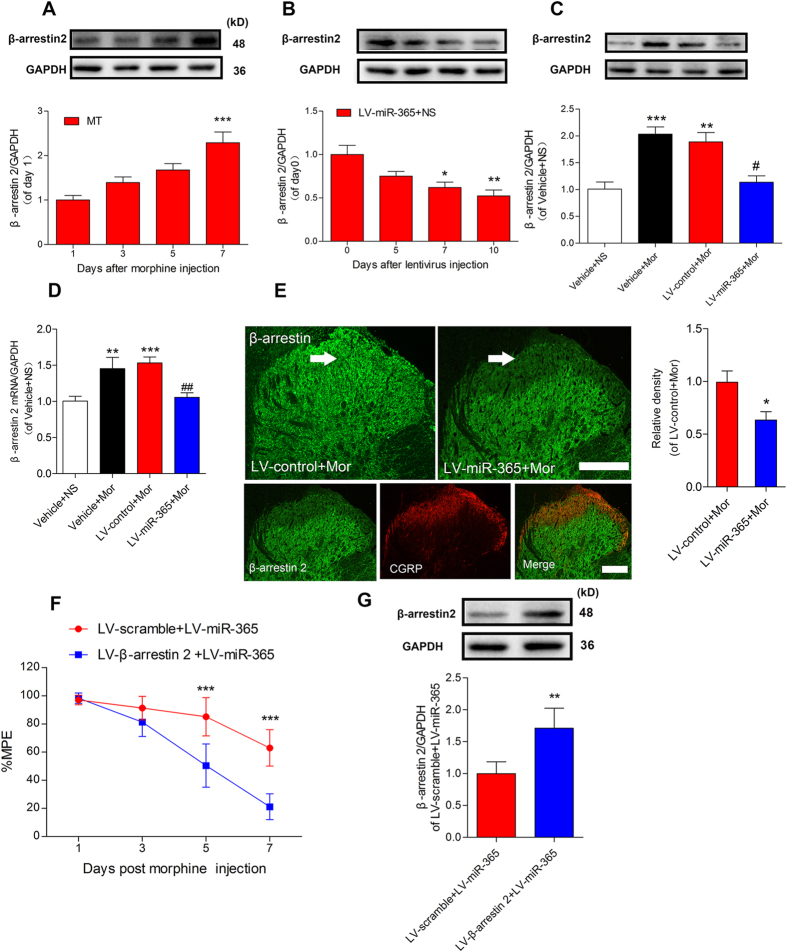
β-arrestin 2 is specifically downregulated in the spinal cord after overexpression of miR-365. (**A**) Expression level of β-arrestin 2 in the L4 ~ 5 spinal cord during development of morphine tolerance. n = 5, ***P < 0.001 versus 1 day after morphine injection by one-way ANOVA followed by Bonferroni test. (**B**) Time course of β-arrestin 2 expression levels after viral transduction in the group of rats treated with LV-miR-365 + NS. n = 5, *P < 0.05, **P < 0.001 compared with 0 day just before lentivirus injection by one-way ANOVA followed by Bonferroni test. (**C**) Expression level of β-arrestin 2 protein in the spinal cord 10 days after induction with the lentiviral vector. n = 5, **P < 0.01, ***P < 0.001 compared with the Vehicle + NS, ^#^*P* < 0.05, compared with LV-control + Mor group by one-way ANOVA followed by Bonferroni test. (**D**) Expression level of β-arrestin 2 mRNA in the L4 ~ 5 spinal cord 10 days after lentiviral vector induction. n = 6. **P < 0.01, ***P < 0.001, compared with the Vehicle + NS group, ^##^*P* < 0.01, compared with the LV-control + Mor group by one-way ANOVA followed by Bonferroni test. (**E**) Immunofluorescent staining for β-arrestin 2 in the dorsal horn of the spinal cord 10 days after miR-365 lentiviral vector injection. Immunofluorescent density of β-arrestin 2 (green) from the morphine treated group was downregulated (especially in the superficial lamina where CGRP is expressed) after injection of LV-miR-365. n = 4, *P < 0.05, compared with the LV-control + Mor group by Student’s *t* test. Scale bar = 200 μm. (**F**) Overexpression of β-arrestin 2 blocked miR-365 mediated attenuation of morphine tolerance. LV-β-arrestin 2 or LV-scramble was co-injected with LV-miR-365 3 days before chronic morphine injection. n = 5, ***P < 0.001 compared with the LV-β-arrestin 2 + LV-miR-365 group by two-way repeated-measures ANOVA followed by Bonferroni test. (**G**) Expression of β-arrestin 2 protein 10 days after co-injection of LV-β-arrestin 2 and LV-miR-365 in morphine treated rats. n = 4, **P < 0.01, compared with the LV-scramble + LV-miR-365 group by Student’s *t* test. All of the data are presented as mean ± s.e.m. miR = microRNA, NS = normal saline, Mor = morphine.

Our luciferase assay results suggest that miR-365 regulates β-arrestin 2 expression levels. Therefore, we determined if expression of β-arrestin 2 was upregulated during development of morphine tolerance. At day 5 and day 7 after morphine injection, β-arrestin 2 was gradually increased (Fig. 5A) and inversely correlated with miR-365 downregulation (Fig. 1E), suggesting that miR-365 and β-arrestin 2 are inversely correlated. To explore the functional relevance between miR-365 and β-arrestin 2, we further examined if miR-365 affects β-arrestin 2 expression levels *in vivo*. In rats with saline injection, the data showed that β-arrestin 2 expression levels gradually decreased after injection of LV-miR-365 (Fig. 5B). Moreover, in rats with morphine infusion, the increased spinal β-arrestin 2 expression was reversed by overexpression of miR-365 but not by the LV-control treatment (Fig. 5C). In addition, the mRNA level of β-arrestin 2 was also decreased in the miR-365 overexpression group (Fig. 5D). Immunofluorescent staining result was in consistence with the immunoblotting results. Furthermore, following double staining showed that the downregulation of β-arrestin 2 was mainly located in the superficial lamina of the dorsal horn, especially in the lamina where CGRP was expressed (CGRP is mainly expressed in the superficial lamina, so it is used as a marker of spinal dorsal horn lamina in the study) (Fig. 5E).

To further explore the role of β-arrestin 2 in miR-365 mediated attenuation of morphine tolerance, rats were simultaneously injected intrathecally with LV-β-arrestin 2 and LV-miR-365 before chronic morphine infusion. Behavioral results suggested that overexpression of β-arrestin 2 abolished miR-365 mediated attenuation of morphine tolerance (Fig. 5F), Moreover, β-arrestin 2 expression remained upregulated after co-injection of β-arrestin 2 and miR-365 overexpression lentivirus vector (Fig. 5G). Taken together, these findings suggest that miR-365 overexpression attenuates morphine tolerance at least partly by decreasing β-arrestin 2 expression.

## Discussion

In our present study, miR-365 was identified as a key functional miRNA for regulation of morphine antinociceptive tolerance. Downregulation of miR-365 is involved in development of morphine tolerance, and exogenous overexpression of miR-365 specifically alleviated morphine tolerance by targeting β-arrestin 2, a key component in MOR desensitization and endocytosis. To our knowledge, this is the first demonstration of a regulatory role for miR-365 in the development of morphine tolerance through regulation of β-arrestin 2. We showed that miR-365 plays a vital role in morphine tolerance, ranging from molecular changes in cells to behavioral changes in animals.

Studies reported that miR-365 is upregulated in human breast cancer^34^, and involved in carcinogenesis of small lung cancer and colon cancer^35^,^36^. Moreover, miR-365 also plays a role in the CNS, in which miR-365 is overexpressed in the rat hippocampus after status epilepticus induced by amygdala stimulation^29^. A previous study found that miR-365 was upregulated after long-term morphine treatment in the mouse hippocampus and primary cultures of rat hippocampal neurons^37^. However, miR-365 has not been linked to morphine tolerance in the study. The data of our study showed a new role of miR-365 in regulating morphine tolerance.

After inducing lentiviral vector-mediated overexpression of miR-365, we found that overexpression of miR-365 specifically suppressed and reversed morphine tolerance. However, we observed that lentiviral vector-mediated overexpression of miR-365 did not fully restore the analgesic effect of morphine, although the expression of miR-365 was robustly increased. Maybe this is because miR-365 only exerts its regulatory effects at the post-transcription level and is not involved in gene transcription. The data from staining showed that miR-365 was expressed in the superficial lamina of the spinal cord dorsal horn where MORs are most abundant. Recent studies have demonstrated that desensitization and internalization of MOR are responsible for the analgesia and antinociceptive tolerance of morphine^38^,^39^,^40^. In our study, β-arrestin 2 was identified as a target gene of miR-365. After LV-miR-365 induction, spinal β-arrestin 2 protein expression and mRNA levels were both down-regulated in a time-dependent manner. The decreased level of β-arrestin 2 mRNA may be attributed to overexpression of miR-365, which cleaved the 3′-UTR of β-arrestin 2 after partially binding to it.

Immunochemistry data also revealed that β-arrestin 2 was down-regulated, especially in the superficial lamina of the dorsal horn. As MOR are most abundant in the superficial lamina, the data indicate that downregulation of β-arrestin 2 may influence MOR function after miR-365 overexpression. β-arrestin 2 is a member of the arrestin protein family, which participates in GPCR desensitization and causes specific cellular responses to stimuli such as neurotransmitters, hormones, and sensory signals^33^,^41^,^42^,^43^. Lefkowitz *et al*. have found that functional deletion of β-arrestin 2 in mice resulted in enhanced and prolonged antinociception in the hot plate test after morphine treatment^30^. Furthermore, the animals did not develop tolerance after acute or chronic morphine treatment, yet they still developed morphine dependence^44^. It has been reported that knockdown of β-arrestin 2 by intrathecal delivery of antisense or siRNA also attenuated morphine tolerance in rats^32^,^45^. The data show that MOR desensitization may be impaired due to the lack of β-arrestin 2, whereas morphine dependence can be dissociated from desensitization. Coincidently, as MOR agonists produce analgesic tolerance, β-arrestin 2 is significantly upregulated in specific brain areas such as the locus coeruleus, cortex, and striatum^31^,^46^,^47^. In the periaqueductal gray (PAG), β-arrestin 2 expression was significantly decreased after chronic morphine treatment and overexpression of β-arrestin 2 abolished antinociceptive effects of morphine^48^,^49^.

Compared with direct inhibition of β-arrestin 2, miRNA modulation may have advantages. First, unlike RNAi or other artificial manipulations, miRNA modulation can reduce the risk of nonspecific off-target silencing. This is because proper miRNA-mRNA interactions have evolved for a billion years. Second, compared with siRNAs or shRNAs that only target a single transcript, miRNAs can target multiple transcript pathways, which may reduce resistance of mutated clones in disease since many simultaneous mutations would be required to subvert the effects of miRNA expression^50^. Therefore, miR-365 targeting of β-arrestin 2 is a suitable and promising approach for treatment of morphine tolerance.

In addition, it has been reported that miR-365 regulates expression of other target genes. Recent studies found that miR-365 could inhibit vascular smooth muscle cell proliferation by down-regulating cyclin D1 and regulate Mycobacterium tuberculosis-induced active pulmonary tuberculosis via interleukin 6 expression^51^,^52^. Interleukin 6 is involved in regulation of the immune response, inflammation, and glial activation^53^. Chronic morphine treatment activates spinal glia and enhances transcription of interleukin 6, and anti-interleukin antibodies can reverse development of morphine tolerance^54^.

Recent studies have demonstrated that there are co-regulatory relationships among miRNAs^55^. Clustered or homologous miRNAs (members of the same miRNA gene family) have close functional relationships and show the same types of deregulation patterns in multiple biological processes^56^. Previous studies have reported that multiple miRNA-based pathways, such as let-7 and miR-23b, contributed to morphine tolerance^10^,^27^, and some of these show the same deregulation pattern of miR-365. However, none of these miRNAs is from the same gene family as miR-365. Because the target genes of let-7 (MOR) and miR-23b (MOR1) interact with the target gene of miR-365 (β-arrestin 2), we cannot rule out that there may be a relationship between these miRNAs and miR-365. Further studies will be required to explore the specific interaction of these miRNAs and miR-365.

We initially considered interleukin -6 and β-arrestin 2 as target genes of miR-365. However, our *in situ* hybridization data showed that miR-365 was located in neurons of spinal cord, so we focused on β-arrestin 2 as a target of miR-365. We cannot exclude the possibility that other target genes of miR-365 are involved in miR-365-induced attenuation of morphine tolerance. Further insight into the role of miR-365 in neurons will enhance our understanding of the molecular mechanisms of morphine tolerance.

**Figure 6:**
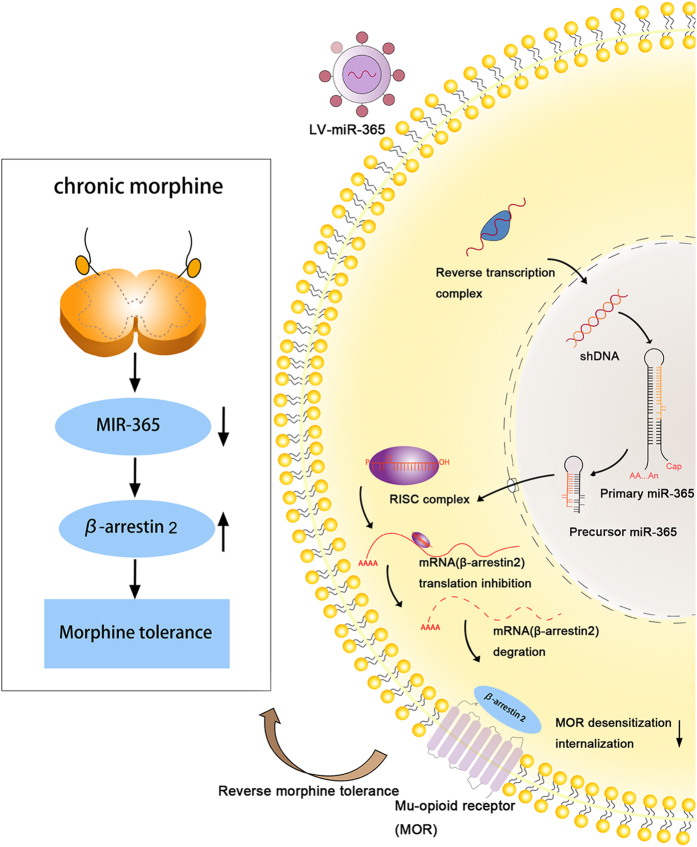
Schematic diagram for the proposed mechanism of morphine tolerance regulation by miR-365. Left panel: Chronic morphine infusion sets up a cascade of events that lead to decreased levels of miR-365 and increased expression of β-arrestin 2, which ultimately causes morphine tolerance. Right panel: After a host cell is infected with a lentivirus, the viral genome is integrated into the host genome in the nucleus to yield primary-miR-365. Primary-miR-365 is processed to yield premature-miR-365, which is exported to the cytoplasm where mature miR-365 is generated and incorporated into the RISCs that are programmed by microRNA. This leads to inhibition of expression of host mRNAs (β-arrestin 2) in the cytoplasm of the infected cell. Decreased expression of β-arrestin 2 attenuates morphine tolerance. RISC = RNA-induced silencing complex, miR = microRNA.

Based on our data, we propose the following pathway. After chronic morphine infusion, miR-365 is downregulated, which alleviates translational repression of its target gene β-arrestin 2 and increases its expression. Upregulated β-arrestin 2 associates with MOR and promotes MOR desensitization and internalization, which contributes to development of morphine tolerance (Fig. 6). After injection of the lentiviral vector- mediated miR-365, upregulated miR-365 was incorporated into an RNA-induced silencing complex (RISC) that mediated translational inhibition. β-arrestin 2 mRNA was then recruited to the RISC, binding to miR-365 with its 3′-UTR. The argonaute protein in the RNA-induced silencing complex destabilized β-arrestin 2 mRNA and repressed its expression. The downregulation of β-arrestin 2 then relieved MOR desensitization and alleviated morphine tolerance.

The current study is the first to demonstrate that miR-365 prevents and reverses development and maintenance of morphine tolerance through regulation of GPCR binding protein β-arrestin 2. However, this study has several limitations. The goal of miRNA overexpression is to restore physiological levels of miRNA for optimal function and avoid unwanted side effects of high levels of miRNA^21^. However, neither lentiviral-mediated overexpression of miR-365 nor a miR-365 mimic can satisfy these requirements. We also realized that further experimental investigations such as genetic deletion of miR-365 in the spinal cord would be needed to clarify the regulatory mechanisms of miR-365. Furthermore, clinical trials will be needed to test whether the findings of the study can be applied to humans.

In conclusion, our results reveal that miR-365 is involved in morphine tolerance by regulating expression of β-arrestin 2. Overexpression of miR-365 results in decreased expression of the target gene β-arrestin 2 and attenuation of morphine tolerance. Overexpressing miR-365 using lentivirus-miR-365 might provide a promising and novel approach to treatment of morphine tolerance. Further studies are required to clarify the distinct role, therapeutic impacts, and potential side effects of miR-365 modulation of morphine tolerance in humans.

## Materials and Methods

### Animals

Male Sprague-Dawley rats (250~300 g) were purchased from the Animal Service of Central South University. All animals were housed in standard transparent plastic cages under standard room conditions (controlled constant temperature of 20 ± 0.5 °C, 12 h:12 h light/dark cycle, with free access to food and water). All experimental procedures were approved by the Animal Care and Use of Central South University. The study adhered to the Ethical Guidelines of the International Association for the Study of Pain^57^. All efforts were made to minimize suffering and number of animals used. Animals were randomly assigned to groups. All animal behavioral tests were performed and assessed by an independent observer blinded to the treatment to minimize bias, and the estimation of sample size was based on previous studies^58^.

For intrathecal catheter implantation, animals were anaesthetized by chloral hydrate (400 mg/kg, IP) and implanted with PE-10 catheters into the intrathecal lumbar (L3 ~ 4) space as previously described^59^. The exterior end of the catheter was sealed by exposure to a mild flame. After catheter implantation, the animals recovered for 3 days before any further experimental manipulations. Rats with motor weakness or paralysis were excluded from further experimentation.

To construct the animal model of morphine tolerance, rats were repeatedly administered morphine (10 μg, i.t.) or saline (10 μL, i.t.) twice a day for 7 days^60^. The antinociceptive effect of morphine was evaluated by the tail flick test. Rats were killed on days 1, 3, 5, and 7 after intrathecal injection.

### Behavioral test

The development and maintenance of morphine antinociceptive tolerance was assessed by the tail flick test as previously described^32^. The tail flick test was performed every other day before and 30 min after morphine or saline injection. Three trials were performed with 3 min intervals and with changes of tail position for each trial. The mean latency from 3 trials was defined as the tail flick latency. Antinociception was calculated as %MPE, where %MPE = (test latency − predrug latency)/(cutoff time-predrug latency) × 100. To prevent tissue damage, the cutoff time was set to 10 s. Antinociceptive responses were presented as mean ± s.e.m. of %MPE^61^.

### miRNA microarray

Total RNA of the L4 ~ 5 lumbar spinal cord was isolated using a miRNAeasy mini kit (QIAGEN). RNA quantity and quality was measured by a spectrophotometer (ND-1000, NanoDrop). The miRNA microarray was performed as described previously^62^. In brief, total RNA (500 ng) was 3′-end-labeled with Hy3^TM^ fluorescent label and hybridized to the miRCURY^TM^ LNA Array (v.16.0, Exiqon). The feature extraction software (Agilent Technologies) was used to quantify the fluorescent intensity of each spot of microarray images, and signal intensities >10 were considered positive expression. The statistical significance of upregulated or downregulated miRNAs were analyzed by t-test. MEV software (v4.6, TIGR) was used to perform hierarchical clustering. We used the Benjamini and Hochberg procedure to control the False Discovery Rate (FDR) in these tests^63^.

### Quantitative real-time PCR

Ten nanograms of total RNA was reverse-transcribed with miRNA-specific primers for microRNA using TaqMan microRNA Reverse Transcription kit (Applied Biosystems) according to the manufacture’s protocol. The miRNA was reversed transcribed by incubation at 16 °C for 30 min, 42 °C for 40 min, 85 °C for 5 min, and held at 4 °C to stop the reaction. Quantitative PCR assays (SYBR Green I master kit, Applied Biosystems) were performed using primers for miR-365 (GSP: GGGTAATGCCCCTAAAAAT, R: CAGTGCGTGTCGTGGAG) with the following conditions: 50 °C for 2 min, 95 °C for 10 min, 40 cycles of 95 °C for 10 s, and 60 °C for 60 s on a 7300HT thermocycler (Applied Biosystems). U6 was used for normalization (Forward: 5′-GCTTCGGCAGCACATATACTAAAAT-3′, Reverse: 5′ CGCTTCACGAATTTGCGTGTCAT-3′).

For detecting β-arrestin 2 mRNA expression, PCR amplifications were performed at 94 °C for 2 min, followed by 94 °C for 30 s, 50 °C for 30 s, and 72 °C for 90 s using primer pairs for β-arrestin 2 min, Forward: 5′-AGCACCGCGCAGTACAAGT-3′, Reverse: 5′-CACGCTTCTCTCGGTTGTCA-3′). GAPDH (Forward: 5′-AAGGTGAAGGTCGGAGTCAAC-3′, Reverse: 5′-CATGAGTCCTTCCACGATACC-3′) was used as a loading control. All samples were run in duplicate. The relative expression level of mRNA was calculated by the 2^−ΔΔCt^ method^64^,^65^.

### *In situ* hybridization and immunochemistry

A digoxin-labeled locked nucleic acid (LNA) detection probe for miR-365 was purchased from Exiqon. After intracardial perfusion with 0.1 mol/L PBS followed by 4% paraformaldehyde in PBS, the lumbar spinal cord (L4 ~ L5) was dissected, and the tissues were post-fixed at 4 °C for 5 h. The tissues were then were immersed in 30% sucrose/PBS for 24 h. The tissues were cut as cryostat sections of 10 μm and preserved at −80 °C. Hybridization for miRNA was performed as described previously^23^. In brief, tissue cryostat sections were rinsed 3 times, fixed in 4% paraformaldehyde for 10 min, washed 3 times again, and then incubated for 20 min with pepsin solution (100 μL pepsin in 3% citric acid). After pretreatment with pepsin, sections were prehybridized in hybridization buffer for 4 h at 47 °C followed by hybridization with denaturation hybridization buffer containing 2 pmol/L miR-365 or a negative control (scrambled probe) at 48 °C overnight. After the slides were stringently rinsed 3 times for 15 min in pre-warmed 2× SSC at 37 °C and for 20 min in 0.2× SSC at room temperature, the sections were blocked for 1 h with blocking buffer, incubated with mouse anti-digoxigenin antibody (1:500, Roche Diagnostics GmbH, clone 1.71.256) for 2 h, rinsed in PBS, and then incubated with cy3-conjugated goat anti-mouse IgG (1:200, Abcam). For double staining, slides were rinsed in PBS, blocked, and incubated with the following primary antibodies at 4 °C overnight: rabbit anti-GFAP (1:600, Millipore), rabbit anti-Iba1 (1:200, Wako), and rabbit anti-NeuN (1:500, Millipore). The next day, slides were rinsed 3 times and incubated with Alexa Fluor 488-conjugated secondary antibodies (1:800, Jackson) for 2 h. For detecting LV-miR-365 and β-arrestin 2, we incubated the slides with rabbit anti-GFP (1:200 Abcam), rabbit anti-β-arrestin 2 (1:200 Abcam), mouse anti-NeuN, and mouse anti-CGRP (1:200 Abcam). After rinsing, slides were incubated with secondary antibodies (1:800 Jackson) for 2 h. A Leica Observer Microscope was used to observe and document the slides. Image-Pro PLUS 6.0 was used to analyze the images.

### Luciferase reporter assay

The luciferase reporter assay was performed as described previously^10^. Briefly, the 3′-UTR of β-arrestin 2 (5′-GAGAGGTAGGGGTGGGCAGGACTGAGGTCCACTGCCCTTGCGGGTAGGAGGGTCCCAGCCTCTCCTCCTTCCCCGTTCGTCCACCCGAGATACACACTGGACCCAtcactcgttgaaagtgggcattaATCTTTTGACTTCAGCTCTGCCACCCCAGCCCTGCTCCCTAGGGTGGCAAGCTGTGTACACACCTAAAGTTTTGGGAAGGGAAGGGAACACTGAAAGCAAGGAGTGAATGTAGAGAAAAGGAGTAGAA-3′) was generated by gene synthesis, which contained the seed sequence complementary to miR-365 (5′-GGGCATT-3′), was subcloned downstream of the *Renilla* luciferase reporter gene into the psiCHECK-2 vector (Promega). The recombinant vector was named pYr-Arrb2-3U. The mutated vector (pYr-Arrb2-3U-Del) was constructed by a partial deletion of the miR-365 targeting sequence (5′-GAGAGGTAGGGGTGGGCAGGACTGAGGTCCACTGCCCTTGCGGGTAGGAGGGTCCCAGCCTCTCCTCCTTCCCCGTTCGTCCACCCGAGATACACACTGGACCCATCACTCGTTGAAAGTgtaATCTTTTGACTTCAGCTCTGCCACCCCAGCCCTGCTCCCTAGGGTGGCAAGCTGTGTACACACCTAAAGTTTTGGGAAGGGAAGGGAACACTGAAAGCAAGGAGTGAATGTAGAGAAAAGGAGTAGAA-3′). The pYr-Arrb2-3U vector and pYr-Arrb2-3U-Del vector constructs with or without either the miR-365 mimic (50 nM) or the scrambled control (50 nM) were transfected into HEK293 cells (70% confluence) (Lipofectamine 2000, Invitrogen). After 48 h, cells were lysed and a dual-luciferase report assay system (Promega) was performed to measure *Renilla* and firefly luciferase activity. The activity was then normalized to the firefly luciferase activity. We performed all the experiments in triplicate.

### Lentivirus vector system

Pri-miR-365 and negative control sequences were cloned into the pGCsil-GFP vector (GENECHEM) to generate the lentivirus-mediated miR-365 vector (LV-miR-365) and the lentivirus negative control vector (LV-control). Virus packaging was performed as follows. The pGCsil-miR-365-GFP vector (20 μg) with pHelper 1.0 Vector (packaging plasmid, 15 μg) and pHelper 2.0 vector (envelop plasmid, 10 μg) were cotransfected into HEK293T cells using Lipofectamine 2000 (Invitrogen). After 48 h of transfection, the culture medium was collected, concentrated by ultracentrifugation, aliquoted, and then stored at −80 °C. The final titer of lentivirus was 3 × 10^8^ TU/mL. LV-β-arrestin 2 and LV-scramble control were purchased from Genepharma with a titer of 5 × 108 TU/ml. In each instance, 10 μL of lentivirus was intrathecally injected *in vivo*.

### Western blot analysis

Western blot was performed as previously described^58^. In brief, protein samples were separated on a SDS-PAGE gel and transferred to a polyvinylidene fluoride membrane (250 mA, 50 min). The blots were blocked with primary antibody for β-arrestin 2 and incubated overnight at 4 °C. The concentration of the primary antibody for β-arrestin 2 was 1:1000 (Abcam), and the concentration of the secondary antibody was 1:5000 (Chemicon). The antibodies were visualized using Western Blot Chemiluminescence Reagent Plus (Millipore). The blots were scanned and quantified using Quantity One analysis software (Bio-Rad) for densitometric analysis. Original uncropped westerns are provided in Supplementary Fig. 1.

### Statistical analysis

No statistical methods were used to predetermine sample sizes. Values are expressed as mean ± s.e.m. There were no data missing in the study. SPSS Version 19.0 (IBM) was used for statistical analyses. A 2-tailed Student’s *t* test was employed for a simple comparison of 2 groups. Multiple comparisons were accomplished using a one-way ANOVA or a two-way ANOVA followed by Bonferroni correction. All tests were 2-tailed, and *P* values less than 0.05 were considered statistically significant.

## Additional Information

**How to cite this article**: Wang, J. *et al*. miR-365 targets β-arrestin 2 to reverse morphine tolerance in rats. *Sci. Rep.* **6**, 38285; doi: 10.1038/srep38285 (2016).

**Publisher's note:** Springer Nature remains neutral with regard to jurisdictional claims in published maps and institutional affiliations.

## Supplementary Material

Supplementary Information
