# Supplementary material for: miR-365 targets β-arrestin 2 to reverse morphine tolerance in rats
Source: Sci Rep. 2016 Dec 6;6:38285. doi: 10.1038/srep38285 (PMC5138852; doi:10.1038/srep38285)
Supplement: Supplementary Information [file srep38285-s1.pdf]

Supplementary Information for

miR-365 targets β-arrestin 2 to reverse morphine tolerance in rats

Jian Wang[1], Wei Xu[1], Tao Zhong[1], Zongbin Song[1], Yu Zou[1], Zhuofeng Ding[1], Qulian Guo[1], Xinzhong Dong[2,3], Wangyuan Zou[1*]

1. Department of Anesthesiology, Xiangya Hospital, Central South University, Changsha, Hunan 410008, China
2. The Solomon H. Snyder Department of Neuroscience, Johns Hopkins University, School of Medicine, Baltimore, Maryland 21205, USA.
3. Howard Hughes Medical Institute, Johns Hopkins University School of Medicine, Baltimore, Maryland 21205, USA.

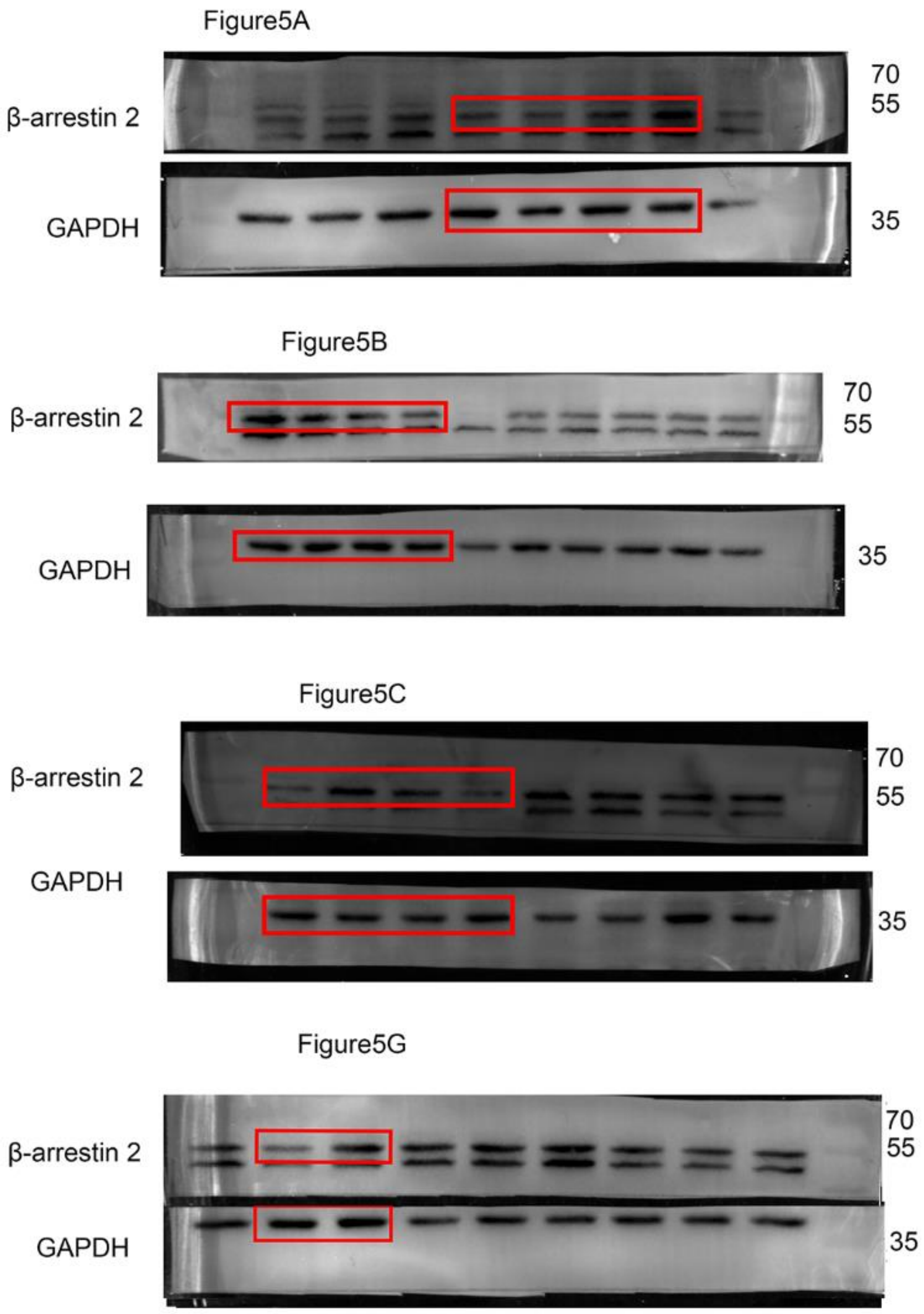

Supplementary Figure 1: original western Blots. Red box highlight area presented in corresponding figures (Figure 5).
